# Endovascular Treatment of Giant Intracranial Aneurysms

**DOI:** 10.7759/cureus.8290

**Published:** 2020-05-26

**Authors:** Italo Linfante, Vincenzo Andreone, Natalia Ravelo, Amy K Starosciak, Bilal Arif, Hussain Shallwani, Peter Tze Man Kan, Michael W. McDermott, Guilherme Dabus

**Affiliations:** 1 Neurology, Miami Cardiac & Vascular Institute/Miami Neuroscience Institute, Baptist Health South Florida, Miami, USA; 2 Neurology & Stroke Unit, Ospedale Antonio Cardarelli, Napoli, ITA; 3 Neuroscience, Herbert Wertheim College of Medicine, Florida International University, Miami, USA; 4 Neurology, Miami Neuroscience Institute, Baptist Health South Florida, Miami, USA; 5 Neurosurgery, University at Buffalo - The State University of New York, Buffalo, USA; 6 Neurosurgery, Baylor College of Medicine, Houston, USA; 7 Neurosurgery, University of California San Francisco, San Francisco, USA; 8 Radiology, Miami Cardiac & Vascular Institute/Miami Neuroscience Institute, Miami, USA

**Keywords:** coiling, endovascular, giant intracranial aneurysm, pipeline embolization device

## Abstract

Objective

Giant intracranial aneurysms (GIAs) are associated with a high risk of rupture and have a high mortality rate when they rupture (65-100%). The traditional microsurgical approach to secure these lesions is challenging, and as such endovascular embolization has been increasingly selected as a treatment option.

Methods

We performed a retrospective analysis of consecutive patients with ruptured and unruptured GIAs at three medical centers from October 2008 to April 2016. Clinical follow-up and digital subtraction angiography were conducted at six months post-treatment. Chi-square analysis was used to determine differences in outcomes between anterior and posterior circulation aneurysms and if a pipeline embolization device (PED) provided favorable outcomes in unruptured GIAs.

Results

A total of 45 consecutive patients (mean/median age = 57/59; range: 16-82 years) were included. The mean/median aneurysm size was 29.9/28.3 mm (range: 25-50 mm). Eight (18%) patients presented with aneurysmal subarachnoid hemorrhage and 37 (82%) with unruptured GIAs. Twenty-eight (62%) were treated with a PED: 11 (24.4%) with one PED, 1 (2.2%) with PED + coils, 11 (24.4%) with more than one PED, and 5 (13.5%) with multiple PED + coils. The overall mortality rate was 3/45 (6.7%). No deaths were procedure-related. Five (11.1%) patients experienced ischemic stroke but only 2 had a 90-day modified Rankin Scale (mRS) score of ≥3. Of 33 patients available for six-month angiography, Raymond scale (RS) scores were 1, 2, and 3 for 23/45 (70%), 7/45 (20.9%), and 3/45 (9.1%), respectively. Chi-square test demonstrated that overall, anterior circulation GIAs had better clinical (mRS score) and radiographic (RS score) outcomes than posterior GIAs. PED alone provided similar clinical mRS outcomes but had a higher rate of complete occlusion at six months compared with PED + coils and coils alone in unruptured GIAs (p < 0.05).

Conclusions

Endovascular embolization using PED or PED + coils appears to be a moderately safe and effective treatment option for patients with GIAs.

## Introduction

Cerebral aneurysms are defined as giant when their diameter is ≥25 mm [1-6]. They represent <5% of all intracranial aneurysms, making them rare among all aneurysms [3-5,7]. The natural history of giant intracranial aneurysms (GIAs) is characterized by progressive growth, thrombosis, and rupture [1,3,5,6,8,9]. Rarely, GIAs present with hemorrhage; therefore, many are revealed by neuroimaging performed for headache, seizure, stroke, or progressive focal deficit [7]. The risk of rupture (RR) of untreated, unruptured GIAs is 8-10% per year, with a mortality of 65-100% at one to five years follow-up [10,11]. For aneurysms of any size, the RR is increased for posterior circulation aneurysms compared with anterior circulation aneurysms; therefore, it is likely that posterior circulation GIAs have a higher RR than anterior circulation GIAs [12]. With these statistics in mind, surgical and endovascular management of patients harboring these lesions is the most appropriate option in most cases.

The treatment of ruptured or unruptured GIAs is particularly challenging, with mortality rates in microsurgical clipping of both ruptured and unruptured GIAs reported to be 6-22% [6,10]. An endovascular approach including parent artery occlusion, coil embolization, stent-coil embolization, remodeling technique, and covered stent has been used more recently as an emerging technique to treat these lesions [6].

Recently, high rates of complete aneurysm occlusion have been reported in the treatment of giant aneurysms with the use of endoluminal flow diverters. The Pipeline Embolization Device (PED; Medtronic, Minneapolis, MN, USA) received FDA approval in 2011 (PMA P100018) for the treatment of large and giant wide neck aneurysms of the internal carotid artery. In the International Retrospective Study of the Pipeline Embolization Device (IntrePED), 66 aneurysms (7.3%) were giant, whereas in the Pipeline for Uncoilable or Failed Aneurysms (PUFS) study, 22 (20.4%) were larger than 25 mm in maximum dimension [2,13]. We present here our recent three-center experience with the endovascular treatment of patients with giant aneurysms using PED, coil, or onyx embolization.

## Materials and methods

The current analysis was approved by Institutional Review Boards of three medical centers: Miami Cardiac & Vascular Institute (MCVI) in Florida, University at Buffalo (UB) in New York, and Baylor College of Medicine (BCM) in Texas. Informed consent for the procedure was obtained from each patient. The patients included in this analysis were obtained from a retrospective review of our database of consecutive ruptured and unruptured GIAs treated endovascularly at our three centers between October 2008 and April 2016.

The decision on the best treatment approach for each was discussed between the cerebrovascular neurosurgical attending and interventional neuroradiology attending. All patients underwent a conventional digital subtraction angiography (DSA) including three-dimensional reconstructed images at the time of admission. Treatment of the aneurysm was based on each operator’s preference, including the timing of external ventricular drain placement, number of PEDs used, coil selection, administration and dose of anti-platelet agents such as aspirin, clopidogrel, and glycoprotein IIb/IIIa inhibitors, and perioperative management of the patients. The timing and modality of follow-up postoperative imaging were also dependent on the operator’s preference and the clinical status of the patient. However, all patients underwent a DSA at six months post-treatment.

We collected basic patient demographics, aneurysm size, location, the number and sizes of PEDs used, use of coiling, 90-day modified Rankin Scale (mRS) score, and follow-up imaging data (Raymond Scale, RS). Position and immediate angiographic result were recorded with conventional DSA imaging with or without XperCT (Philips Healthcare, Best, the Netherlands) and Low Contrast Imaging (LCI, Canon Medical Systems,Tochigi, Japan). Patients were admitted to the intensive care unit (ICU) following treatment for further observation and management.

The primary clinical outcome was the 90-day mRS score and the primary radiographic outcome was the six-month RS score. Rate of follow-up for clinical and radiographic outcomes and the outcomes themselves were compared for anterior and posterior circulation aneurysms. Because of the nature of the individualized therapy provided for each case, there was a relatively small sample size in treatment types, such as PED alone versus PED + coils in anterior and posterior or ruptured and unruptured aneurysms. Therefore, in unruptured aneurysms only, chi-square analysis was used to determine if proportions of good clinical (mRS score: 0-2 vs. 3-6) and good radiographic (RS score: 1 vs. 2-3) outcomes were statistically significantly different between those treated with PED only, PED + coils, and coils alone.

## Results

Data were collected on a series of 45 consecutive patients with a diagnosis of ruptured or unruptured giant aneurysm who were treated endovascularly from October 2008 to April 2016 (Table 1). Of the 45 patients, 15 were treated at MCVI, 21 at UB, and 9 at BCM. The study population consisted of 30 (66.7%) females and 15 (33.3%) males, with a mean age of 57 years (median: 59 years; range: 16-82 years).

**Table 1:** Summary of cases H&H, Hunt & Hess Scale; mRS, modified Rankin Scale; RS, Raymond Scale; L, left; SCA, superior cerebellar artery; ICA, internal carotid artery; MCA, middle cerebral artery; PED, pipeline embolization device; R, right; VA, vertebral artery; IA, intra-arterial; TIA, transient ischemic attack; SAH, subarachnoid hemorrhage; IPH, intraparenchymal hemorrhage; BA, basilar artery; ASA: acetylsalicylic acid; IV, intravenous; n-BMA, n-butyl methacrylate; CN, cranial nerve; PCOM, posterior communicating artery.

Case	Age	Location	Size (mm)	Ruptured unruptured	H&H scale	Initial treatment	Complication	mRS score pre-procedure	90-day mRS score	Rescue therapy	Six-month RS
1	80s	L SCA	32	Unruptured	n/a	Onyx embolization	None	1	1	None	1
2	50s	L ICA	28	Ruptured	III	Coiling	None	0	0	None	1
3	60s	L MCA	25.3	Ruptured	I	Coiling	Secondary ischemic stroke	0	0	Flow diverter for residual aneurysm lumen	1
4	50s	L ICA	25	Unruptured	n/a	PED	None	0	0	None	1
5	60s	L MCA	26	Unruptured	n/a	PED	Mild vasospasm	0	0	None	1
6	60s	R VA	35	Unruptured	n/a	Coiling	None	4	6	None	n/a
7	60s	L ICA	25.2	Unruptured	n/a	PED	None	1	0	None	1
8	70s	L ICA	40	Unruptured	n/a	PED	None	2	2	None	1
9	60s	R ICA	46	Unruptured	n/a	PED	None	1	1	None	1
10	50s	L ICA	26	Unruptured	n/a	Coiling	None	5	2	Endovascular treatment for residual aneurysm lumen	1
11	Teen	L ICA	28.1	Unruptured	n/a	PED	None	0	1	None	1
12	40s	R MCA	25	Ruptured	III	Coiling	Secondary ischemic stroke	4	1	Hemicraniectomy for hydrocephalus, IA nicardipine and angioplasty of the supraclinoid ICA for vasospasm	1
13	70s	L ICA	25	Unruptured	n/a	Coiling	None	0	0	None	1
14	20s	R MCA	26	Ruptured	IV	Coiling	Intracranial bleeding	5	6	None	n/a
15	60s	R ICA	32	Unruptured	n/a	PED	None	1	0	None	1
16	60s	L ICA	28	Unruptured	n/a	PED	None	1	0	None	n/a
17	50s	R ICA	50	Unruptured	n/a	PED (x2) and coiling	Femoral artery pseudo-aneurysm	0	0	Evacuation of hematoma from femoral artery pseudoaneurysm through drain	n/a
18	50s	R ICA	27	Unruptured	n/a	PED	None	1	1	Two additional PEDs for residual aneurysm	3b
19	60s	R ICA	26	Unruptured	n/a	PED (x2)	Intraoperative TIA; post-operative SAH and IPH	2	4	Integrilin (for TIA), craniectomy, and clot evacuation (for SAH and IPH); two additional PEDs for residual aneurysm	1
20	50s	R ICA	31	Unruptured	n/a	PED	None	1	0	None	1
21	50s	L ICA	35.8	Unruptured	n/a	PED (x3)	None	1	6 (not procedure related)	None	n/a
22	60s	R ICA	28.5	Unruptured	n/a	PED (x2) and coiling	None	1	n/a	None	n/a
23	40s	BA	37.1	Unruptured	n/a	PED (x3) and coiling	Intraoperative perforator occlusion (causing TIA)	1	n/a	IA Integrilin	n/a
24	30s	R MCA	25.3	Unruptured	n/a	PED (x2) and coiling	Complete PED occlusion, and right lentiform and caudate nucleus infarct	0	1	Integrilin, heparin, additional doses of ASA and clopidogrel, attempted aspiration thrombectomy of MCA embolic material	1
25	50s	R ICA	30	Unruptured	n/a	PED (x2)	None	1	0	None	1
26	40s	R ICA	27.7	Ruptured	III	Stent-assisted coiling	None	3	n/a	Multiple endovascular treatments for residual aneurysm	n/a
27	60s	L ICA	28.9	Unruptured	n/a	PED	Incomplete coverage of aneurysm by PED	0	n/a	Multiple PED deployment (delayed)	n/a
28	60s	R ICA	27.4	Unruptured	n/a	PED (x3)	Mild cavernous sinus syndrome	1	1	Prednisone	1
29	30s	BA	36	Unruptured	n/a	Multiple stents for vessel reconstruction	Thrombosis of small perforators, subsequent reperfusion hemorrhage	1	n/a	Anticoagulation and IV Integrilin for perforator thrombosis	n/a
30	50s	L ICA	25	Ruptured	IV	Balloon-assisted coiling	Bilateral ICA vasospasm (unrelated to procedure)	5	n/a	IA verapamil for bilateral ICA vasospasm; multiple endovascular treatments for residual aneurysm	n/a
31	40s	R ICA	30	Unruptured	n/a	Aneurysm neck coiling and parent vessel sacrifice (coiling and n-BMA embolization)	Right CN VII (peripheral) plegia	1	n/a	None	n/a
32	40s	R PCA	26.3	Unruptured	n/a	Stent-assisted coiling	L ICA and MCA vasospasm and ischemic stroke	1	1	Balloon angioplasty for L ICA and MCA vasospasm; IA verapamil for L ICA; multiple endovascular treatments for residual aneurysm	2
33	40s	R ICA	26.4	Ruptured	IV	Coiling	Secondary ischemic stroke	5	3	IV Integrilin (for ischemic stroke); multiple endovascular treatments for residual aneurysm	3b
34	60s	R ICA	25	Unruptured	n/a	Balloon-assisted coiling	None	1	2	Multiple endovascular treatments for residual aneurysm	2
35	40s	L ICA	29	Unruptured	n/a	Stent-assisted coiling	None	1	1	Repeat endovascular treatment for residual aneurysm	3a
36	70s	L ICA	25.8	Ruptured	III	Delayed stent-assisted coiling	None	0	0	Multiple endovascular treatments for residual aneurysm	2
37	40s	R PCA	30	Unruptured	n/a	PED (x3)	In-stent stenosis at 3 months, complete stent occlusion at 6 months, but asymptomatic	0	0	None	1
38	80s	BA + L VA	30	Unruptured	n/a	PED (x3) + coil occlusion of R VA	Secondary ischemic stroke	3	4	None	2
39	70s	BA	40	Unruptured	n/a	Enterprise followed by 2 PEDs + coils	None	0	0	None	2
40	30s	BA	30	Unruptured	n/a	1 PED and 1 enterprise distally, coils to occlude L VA	Cortical PCA vasospasm	3	3	At 3 months, PED foreshortened into aneurysm, requiring additional PED	1 at 9 months
41	60s	L VA	30	Unruptured	n/a	PED (x9)	R hand weakness from L thalamic stroke	3	3	None	2
42	70s	L ICA	30	Unruptured	n/a	PED (x4)	None	0	0	None	1
43	70s	L ICA	25	Unruptured	n/a	PED (x2)	None	2	2	None	1
44	50s	R PCOM	35	Unruptured	n/a	PED (x2) + coils	None	0	0	None	2
45	70s	R ICA	25	Unruptured	n/a	PED (x2)	None	1	0	None	1

Aneurysm characteristics

Mean aneurysm size was 29.9 mm (median/median: 28.1/28.3 mm; range: 25-50 mm). Eight (18%) patients presented with aneurysmal subarachnoid hemorrhage (aSAH). In these cases, the ruptured aneurysms had a mean/median diameter of 26.2/26 mm. In 37 (82%) patients, the aneurysm was discovered as an incidental finding (mean/median diameter: 30.7/30 mm).

Of the aneurysms, 34 (76%) were found in the anterior cerebral circulation and 11 (24%) in the posterior circulation (superior cerebellar artery, vertebral artery [VA], basilar artery, posterior cerebral artery).

Aneurysm treatments

**Figure 1:**
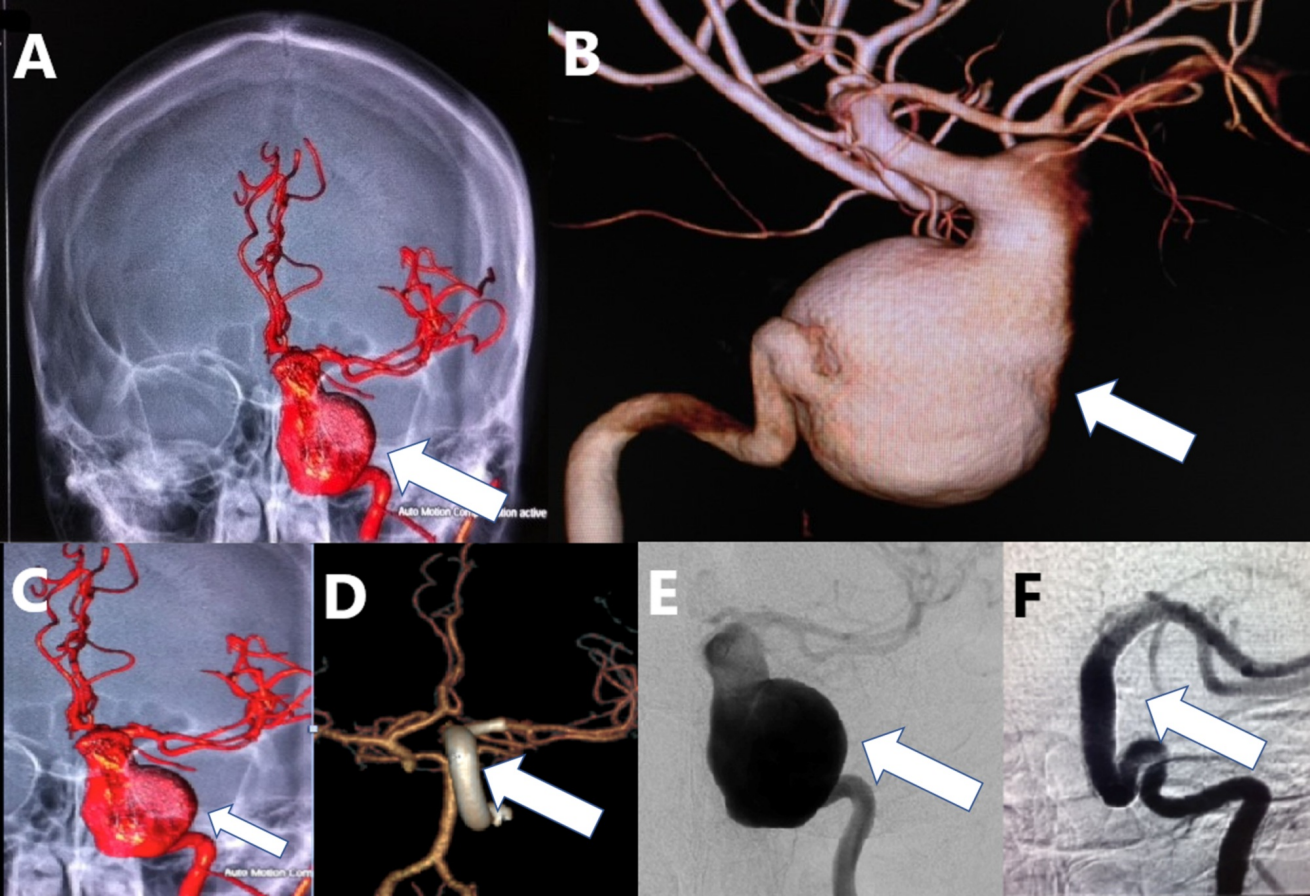
A young patient with an unruptured giant, fusiform, dissecting left ICA aneurysm treated with multiple FDs. (A-C): Three-dimensional reconstruction of digital subtraction angiography of the left ICA contrast injection of the aneurysm (white arrows). (D) The aneurysm was treated by an implant of multiple FDs as a construct from the left middle cerebral artery into the petrous segment of the left ICA (white arrow). (E, F) AP view of the left ICA injection before treatment (E) and one year after treatment with FD (F) demonstrating complete occlusion of the aneurysm and reconstruction of the ICA (white arrows). ICA, internal carotid artery; FD, flow diverter; AP, anterior-posterior

**Figure 2:**
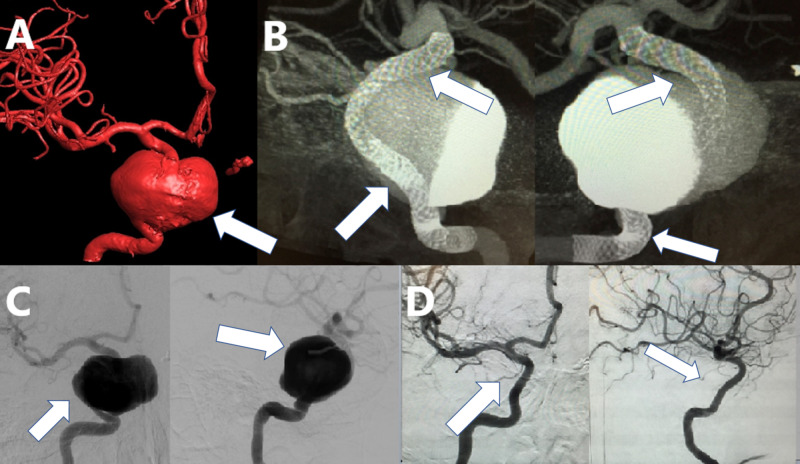
A patient with an unruptured giant cavernous and supraclinoid aneurysm of the right ICA. The aneurysm was treated by implant of a construct of FD devices. (A) Three-dimensional reconstruction of digital subtraction angiography of the right ICA contrast injection of the aneurysm (white arrow). (B) XperCT reconstruction of the right ICA contrast injection after implant of a construct of multiple FD devices from the ICA terminus to the petrous segment of the right ICA (white arrows). (C) Digital subtraction angiography in AP and lateral views before the implant (white arrows). (D) Follow-up angiography in AP and lateral views one year after treatment demonstrating complete occlusion of the lesion with reconstruction of the artery (white arrows). ICA, internal carotid artery; FD, flow diverter; AP, anterior-posterior

Of the 45 giant aneurysms in this series, 28 (62%) were treated with PED: 11 with a single PED (24%) only, 1 with a single PED + coiling (2%), and 16 with more than one PED, with or without coiling (36%). Eight (18%) aneurysms were treated with coiling alone, four (9%) with stent-assisted coiling (including one delayed stent-assisted coiling), and two (4%) with balloon-assisted coiling. The remaining three aneurysms were treated with other treatments including parent vessel sacrifice (coiling and n-butyl methacrylate embolization) (2%), onyx embolization (2%), and multiple stents (2%). Two cases successfully treated with multiple PEDs are shown in Figures 1 and 2.

The distribution of treatments for ruptured aneurysms compared with unruptured aneurysms was quite different. All eight ruptured aneurysms were treated with coiling, and none with PED or other treatments. Five (63%) were treated with coiling alone, 2 (25%) with stent-assisted coiling, and 1(13%) with balloon-assisted coiling. In contrast, only 3 (8%) of 37 unruptured aneurysms were treated with coiling alone, 2 (5%) with stent-assisted coiling, and 1 (3%) with balloon-assisted coiling. Twenty-eight (76%) unruptured aneurysms were treated with PEDs: 21 (57%) with PED only and 7 (19%) with PED + coiling. The remaining three were treated with the other treatments as described in the previous paragraph. In summary, ruptured aneurysms were treated only with coiling or assisted coiling, and unruptured aneurysms were treated primarily with PEDs.

Complications

Mortality

Overall, mortality for this series was 3/45 (7%): one occurred in a patient with aSAH secondary to the giant aneurysm (Hunt and Hess grade IV) and two occurred in unruptured aneurysms. No mortality was procedure-related. In particular, one patient (case 14) was affected by chronic alcoholism and cocaine addiction, who presented with aSAH (Hunt and Hess grade IV) secondary to a ruptured 26-mm right middle cerebral artery (MCA) aneurysm. This aneurysm was treated by coil embolization; however, the patient never recovered to a good neurological status and subsequently developed multiple subarachnoid hemorrhage (SAH) and ICU-related complications and was made comfort care by the family. Another patient (case 6) presented with a 35-mm unruptured right VA aneurysm treated with coil embolization subsequently expired secondary to medical complications related to advanced Parkinson’s disease. The third patient (case 21), with a 35.8-mm unruptured left ICA aneurysm treated with three PEDs, died several months later from causes unrelated to the procedure.

Stroke

Five (11.1%) patients experienced ischemic strokes, two had ruptured aneurysms, and three had unruptured aneurysms. The first one presented with aSAH secondary to a 25.3-mm ruptured MCA aneurysm, was treated with coiling, and needed rescue treatment consisting of a flow diverter for aneurysmal re-growth (case 3). This patient recovered completely from the neurological deficit and had a 90-day mRS score of 0. The second patient presented with aSAH (Hunt and Hess grade IV) secondary to a ruptured 26.4-mm right ICA aneurysm, was treated with coiling, and needed rescue treatment with intravenous Integrilin (case 33). This patient had a 90-day mRS score of 3. Another patient (case 24) presented with a 25.3-mm unruptured right MCA aneurysm was treated with two PEDs and coiling and then had complete PED occlusion and right lentiform and caudate nucleus infarct with clinical worsening (pre-procedure mRS score = 2; 90-day mRS score = 4). The fourth patient had a 30-mm unruptured basilar and left VA aneurysm treated with three PEDs and coiling of the right VA (case 38). The pre-procedure mRS score was 3, which worsened to 4 at 90 days. The fifth patient presented with a 30-mm unruptured left VA aneurysm that was treated with nine PEDs (case 41). A left thalamic stroke occurred causing right hand weakness, but the mRS score at 90 days was 3, which was the same as pre-procedure. A patient who had a 36-mm unruptured basilar tip aneurysm treated with multiple stents had thrombosis of small perforators with subsequent reperfusion hemorrhage (case 29). The patient received anticoagulation and IV Integrilin as rescue therapy. This patient was lost to follow-up before the 90-day mRS score could be obtained.

Follow-up

Clinical Follow-up

At the time of treatment, 14 (31.1%) of 45 patients had no disability (mRS score = 0), 21 (46.7%) had minimal disability (mRS score = 1-2), 4 (8.9%) had moderate disability (mRS score = 3), 2 (4.4%) had moderately severe disability (mRS score = 4), and 4 (8.9%) had severe disability (mRS score = 5).

Thirty-five (82.2%) patients were available for clinical follow-up at 90 days; three had died and seven were lost to follow-up. Thirty (85.7%) patients had good outcomes (90-day mRS score ≤ 2). Three (8.6%) patients had a moderate disability (90-day mRS score = 3). Two (5.7%) of the remaining patients had moderately severe disability (90-day mRS score = 4). Of the 35 patients with 90-day follow-up, there were 2 patients who had some disability that improved from baseline (mRS score of case 10 = 5 to 2; mRS score of case 33 = 5 to 3).

Angiographic Follow-up

Overall, 33/45 (73.3%) patients were available for six-month angiographic follow-up. In addition, 23 (70%) patients had a RS score of 1, 7 (20.9%) had a score of 2, and 3 (9.1%) had a score of 3.

Comparison of Outcomes in Anterior versus Posterior Circulation Aneurysms

Ischemic stroke occurred in 3/34 anterior circulation cases (9%) and 2/11 posterior circulation case (18%). Mortality occurred in 2/34 in anterior circulation (6%) and 1/11 posterior cases (9%). Ninety-day mRS score was available for 29 of 34 anterior cases (85%) and 9 of 11 cases posterior cases (82%), which is a comparable rate of follow-up between groups using the chi-square test (p > 0.05). The proportion of good clinical outcome (mRS score ≤ 2) was 25/29 (86%) for anterior circulation cases and 5/9 (55%) for posterior circulation cases, a difference that was statistically significant (χ2 [1, N = 38] = 3.88; p < 0.05). At six months, the RS was available for 24/34 anterior circulation cases (71%) and 8/11 (73%) posterior circulation cases, which, again, is a comparable rate of follow-up (p > 0.05). The proportion of good angiographic outcome (RS = 1) for anterior circulation aneurysms was 19/24 (79%) compared with 3/8 (38%) for posterior circulation aneurysms. This difference was also statistically significant (χ2 [1, N = 32] = 4.85; p < 0.05). These results are summarized in Table 2.

**Table 2:** Comparison of treatment outcomes in anterior versus posterior circulation PED, pipeline embolization device

		90-day modified Rankin Scale			Raymond Scale		
Aneurysm type	Treatment	Good (0-2)	Poor (3-6)	n/a	Good (1)	Poor (2-3)	n/a
Anterior	PED only	15	2	1	14	1	3
	PED + coil	2	0	1	1	0	2
	Coil only	8	2	2	5	4	3
	Other	0	0	1	0	0	1
Posterior	PED only	2	1	0	1	2	0
	PED + coil	1	2	1	1	2	1
	Coil only	1	1	0	0	1	1
	Other	1	0	1	1	0	1

Comparison of Treatments in Unruptured Aneurysms Only

At 90 days, in patients with unruptured aneurysms who received PED only, 16/19 (84%) had good clinical outcome (mRS score ≤ 2). In comparison, 5/6 (83%) of patients with unruptured aneurysms who received coiling alone had a good clinical outcome and 4/6 (67%) patients who had a PED + coiling had a good clinical outcome. At six months, 15/17 (88%) patients who received PED only had an RS score of 1, 2/5 (40%) patients who received PED + coiling had an RS score of 1, and only 2/5 (40%) of those who received coiling alone had an RS score of 1 (Table 3). Using the chi-square analysis, there was no difference in the proportion of patients with good (mRS score = 0-2) versus poor (mRS score = 3-6) clinical outcomes across treatment groups (p = 0.81). However, the proportion of good (RS score = 1) vs. poor (RS score = 2-3) radiographic outcomes was statistically significantly better for the group treated with PED alone compared with those treated with PED + coiling or coiling alone (χ2 [1, N = 31] = 7.02; p < 0.05).

**Table 3:** Comparison of treatment outcomes in ruptured versus unruptured aneurysms PED, pipeline embolization device

		90-day modified Rankin Scale			Raymond Scale		
Aneurysm type	Treatment	Good (0-2)	Poor (3-6)	n/a	Good (1)	Poor (2-3)	n/a
Ruptured	PED only						
	PED + coil						
	Coil only	4	2	2	3	2	3
	Other						
Unruptured	PED only	16	3	1	15	2	3
	PED + coil	4	2	2	2	3	3
	Coil only	5	1	0	2	3	1
	Other	1	0	2	1	0	2

## Discussion

Our cohort of 45 ruptured and unruptured GIAs treated by the endovascular approach represents a contemporary treatment outcome of these lesions in three medical centers. The overall mortality rate was 6.7% (3/45), with none of them being procedure-related. Five (11.1%) patients experienced ischemic strokes and only two had a 90-day mRS score of ≥3. Of the 35 patients available at 90-day follow-up, 85.7% (30/35) had good clinical outcomes (90 days mRS score ≤ 2), and 2 patients were improved from baseline. Complete aneurysm occlusion was demonstrated in 70% (23/33) of patients at six months follow-up through DSA.

In the literature, ruptured and unruptured GIAs are associated with severe neurological morbidity and mortality. In particular, the RR for untreated, unruptured GIAs was reported to be 8-10% per year, with a mortality of 65-100% at two to five years follow-up [10,11]. When GIAs are treated by microsurgical clipping, the mortality rates of both ruptured and unruptured GIAs were reported at 6- 22% [6,10]. As such, our mortality rate for endovascular treatment of GIAs was better than the rate described for surgical clipping. Several authors reported good clinical outcomes when GIAs were treated by an endovascular approach. In particular, Cekirge et al. reported a complete occlusion rate of 76% post-procedure, 80% at three to six months, and 93% at two to five years in the giant aneurysm group by using liquid embolic agents [14]. However, the authors did not state the rates of good clinical outcomes specifically for the giant aneurysm group. Sluzewski et al. reported good clinical outcomes in 79% of very large and giant aneurysms at a median follow-up at 50 months, though 41% of aneurysms were still incompletely occluded even after repeated coiling [15].

The work on flow diversion technology by Wakhloo and Gounis and by Lieber et al. undoubtedly was a major step forward for the treatment of intracranial aneurysms [16,17]. The safety and efficacy of PED have been demonstrated in numerous studies. Pooled analysis of such studies has shown that PED is associated with a low risk of complications and high angiographic occlusion rates. Nelson et al. showed 93% occlusion rates at six-month angiographic follow-up, with only 6.5% of patients experiencing major neurological complications when treated with PED [18]. In the PUFS study, the mean aneurysm size was 18.2 mm, with 22 (20.4%) of 108 aneurysms being giant. Complete occlusion in the entire cohort was 73.6% and 86.6% at 180-day and one-year follow-up, respectively [2]. Similarly, in our study, complete occlusion was demonstrated in 88% of patients at six months. In the IntrePED study, 66 aneurysms (7.3%) were giant [13]. At 30 days, the morbidity and mortality rates were higher in patients with giant aneurysms (25.8%) compared with large (8.8%) and small (5.4%) aneurysms. The giant aneurysm group demonstrated higher rates of spontaneous ruptures and ischemic strokes when compared with other groups [13]. Similarly, in a meta-analysis of spontaneous aneurysm rupture after flow diversion treatment, the relative RR was 23% for GIAs [19]. Lastly, in a retrospective review that included large and GIAs treated with PED or PED + coils, the authors found complete or near-complete occlusion at the last follow-up in 77% of cases. Of the patients, 12% had symptomatic ischemic complications and 8% had symptomatic hemorrhagic complications, and the overall mortality was 6% [20]. However, there were no separate results for GIAs, nor were comparisons made between large and giant aneurysms. In our study, the rate of ischemic stroke was 5% (1/21) and the mortality rate was 5% (1/21). Our results strengthen the current body of PED literature, which indicates that PED is safe and effective for the treatment of unruptured GIAs.

Coiling alone is a treatment option for GIAs, but it has poor long-term outcomes because giant aneurysms are often incompletely occluded and require repeated coiling [4]. For our ruptured GIAs treated with coiling, we found that 67% had favorable clinical outcomes at 90 days and 60% had complete occlusion at six months. In unruptured GIAs, 83% had favorable clinical outcomes at 90 days, although only 40% exhibited complete occlusion at six months when treated with coiling alone. In comparison, 89% of cases that received any PED treatment (with or without coiling) had a good clinical outcome at 90 days and 92% had complete occlusion at six months. In addition, three of five ischemic strokes were in patients who were treated with coiling only (two ruptured, one unruptured). In the report by Park et al., PED in combination with coiling was shown to result in higher neurological morbidity and required longer procedural time versus PED alone [21]. In our study, there were eight cases of PED with coiling; four of six cases that followed up at 90 days had good clinical outcome, and out of the five follow-ups at six months, only two showed complete occlusion. Stent-assisted coiling and PED has shown to be equally effective, with no significant differences in complications and angiographic outcomes [22]. Interestingly, we found that all three of the cases treated with stent-assisted coiling had incomplete occlusion at six months, even though they all had a good clinical outcome at 90 days. There was one case with a 90-day mRS score of 3 that did not follow-up at six months.

In this series, coiling with or without PED was guided by operator preference. Usually, if possible, coils were placed in the aneurysmal sac. In case of a ruptured GIA, coiling is usually the first approach in order to avoid dual anti-platelet regimen in a patient with an acute SAH.

Overall, our results of PED in unruptured GIAs are similar to those previously reported in the setting of controlled trials [23]. Our current analysis suggests a role of the combination of PED and coils in achieving high occlusion rates. Given the poor natural history of GIAs, a careful study of each patient’s GIA anatomy and relationships with afferent and efferent vessels is recommended to determine the optimum treatment.

Limitations

The present cohort has all the inherent limitations of a retrospective analysis of patients treated endovascularly over an eight-year period. The choice of treatment and follow-up timing were based on the operating physician preference. The perioperative management and choice of rescue treatment were also based on physician preference. Because of these treatment choices, all of the ruptured aneurysms were treated with coiling and none were treated with PED. Therefore, direct comparisons between treatment types in ruptured and unruptured aneurysms could not be made. In addition, no GIAs were treated at these centers with surgical clipping; therefore, comparisons with conventional surgery could not be made. However, angiographic follow-up was consistent in all centers, and patients had conventional DSA at the time of admission and at the six-month follow-up period. Several patients were lost to follow-up by six months. The study does not take into account advances in technology that have occurred in the last eight years. All of the operators from the centers selected for this analysis are high-volume centers for endovascular treatment for complex aneurysms; therefore, these results may not be generalizable to other lower volume centers.

## Conclusions

Our study suggests that endovascular treatment of GIAs either by coil embolization or PED (±coiling) is feasible and can be accomplished with relatively low complication rates and moderate long-term clinical and radiographic outcomes. Future studies with current endovascular technology may be needed to define the best approach for these lesions.
